# Event-based knowledge elicitation of operating room management decision-making using scenarios adapted from information systems data

**DOI:** 10.1186/1472-6947-11-2

**Published:** 2011-01-07

**Authors:** Franklin Dexter, Ruth E Wachtel, Richard H Epstein

**Affiliations:** 1 Department of Anesthesia, University of Iowa, 6JCP, 200 Hawkins Drive, Iowa City, Iowa 52242, USA; 2 Department of Anesthesiology, Jefferson Medical College, #548, 111 S. 11th St., Philadelphia, Pennsylvania, 19107 USA

## Abstract

**Background:**

No systematic process has previously been described for a needs assessment that identifies the operating room (OR) management decisions made by the anesthesiologists and nurse managers at a facility that do not maximize the efficiency of use of OR time. We evaluated whether event-based knowledge elicitation can be used practically for rapid assessment of OR management decision-making at facilities, whether scenarios can be adapted automatically from information systems data, and the usefulness of the approach.

**Methods:**

A process of event-based knowledge elicitation was developed to assess OR management decision-making that may reduce the efficiency of use of OR time. Hypothetical scenarios addressing every OR management decision influencing OR efficiency were created from published examples. Scenarios are adapted, so that cues about conditions are accurate and appropriate for each facility (e.g., if OR 1 is used as an example in a scenario, the listed procedure is a type of procedure performed at the facility in OR 1). Adaptation is performed automatically using the facility's OR information system or anesthesia information management system (AIMS) data for most scenarios (43 of 45). Performing the needs assessment takes approximately 1 hour of local managers' time while they decide if their decisions are consistent with the described scenarios. A table of contents of the indexed scenarios is created automatically, providing a simple version of problem solving using case-based reasoning. For example, a new OR manager wanting to know the best way to decide whether to move a case can look in the chapter on "Moving Cases on the Day of Surgery" to find a scenario that describes the situation being encountered.

**Results:**

Scenarios have been adapted and used at 22 hospitals. Few changes in decisions were needed to increase the efficiency of use of OR time. The few changes were heterogeneous among hospitals, showing the usefulness of individualized assessments.

**Conclusions:**

Our technical advance is the development and use of automated event-based knowledge elicitation to identify suboptimal OR management decisions that decrease the efficiency of use of OR time. The adapted scenarios can be used in future decision-making.

## Background

Operating room (OR) information system data or anesthesia information management system (AIMS) data can be analyzed to identify and quantify potential improvements in performance. For example, McIntosh and colleagues reviewed how to calculate changes in OR allocations to achieve maximal efficiency of use of OR and/or anesthesia time [1]. See Table 1 for definitions. McIntosh et al. also reviewed the potential savings achievable by reducing turnover times [1]. Because such analyses can be (and have been) automated, generation of reports is practical for health systems comprised of multiple facilities.

**Table 1:** Examples of explanations that start each chapter, as described in Section 6, with the reference numbers in brackets changed to this article's reference numbers

**a. Explanation at start of chapter 5: Definitions - OR Efficiency (Table 2). This chapter contains definitions used throughout the article and for the other scenarios**.
Case duration is defined as the time from when a patient enters an OR until he or she leaves the OR.
Turnover time is the time from when one patient exits an OR until the next patient on that day's OR schedule enters the same OR [2,18]. Separating turnover time from case duration permits the two to be studied statistically as separate processes. Turnover times include cleanup times and setup times, but not scheduled delays between cases.
An example of a scheduled delay would be when the first patient of the day is scheduled for surgery that is anticipated to be completed by 11 AM, and the second case of the day in the OR will be performed by a different surgeon who is not scheduled to be available until 1 PM.
Elective OR workload of a surgical service is its total hours of elective cases including turnover times throughout the workday, not just during scheduled OR hours [1].
Under-utilized OR time is the positive difference between allocated OR time and the OR workload [1].
Over-utilized OR time is the positive difference between OR workload and allocated OR time. When allocated OR time and scheduled OR hours are the same (e.g., a service is allocated an OR for 10 hr from 7 AM to 5 PM) and allocated OR time has not been released, then over-utilized OR time is the same as the hours that ORs run past the end of scheduled OR hours [1]. Hourly employees often receive overtime when working during over-utilized hours.
Inefficiency of use of OR time equals the sum of two products: hours of under-utilized OR time multiplied by the cost per hour of under-utilized OR time and hours of over-utilized OR time multiplied by the cost per hour of over-utilized OR time [1]. The cost per hour of over-utilized OR time is invariably more expensive than the cost per hour of under-utilized OR time, in that staff want to get home on time.
OR efficiency is the value that is maximized when the inefficiency of use of OR time has been minimized [1].
**b. Explanation at start of chapter 14: Releasing OR Time Based on OR Efficiency (Table 2). The scenario in **Table 4 **is an example of application of the explanation**.
Occasionally, a service will have filled its allocated OR time and have another case to schedule. Then, OR efficiency is enhanced by scheduling the case into the OR time of the service expected to have the most under-utilized OR time, assuming availability of the surgeon, equipment, etc [1,19,20]. The service "releasing" its allocated OR time is not losing access to OR time, because its surgeons can continue to book cases.
Services fill their allocated OR time at different rates. For example, at one hospital, the median times between when a patient was scheduled for surgery and the actual day of surgery ranged from 2 to 27 days. Whereas outpatient ophthalmology scheduled cases weeks before the day of surgery, cardiac surgery scheduled cases a few days before the day of surgery. Consequently, to maximize OR efficiency, allocated OR time cannot be released for all services the same number of pre-specified days before surgery [1,19].
Predicting which service will have the most under-utilized OR time on the day of surgery is not the same as determining which service has the largest difference between allocated and scheduled OR time when the new case is scheduled. However, the difference in OR efficiency between the two methods is very small. Thus, the service that should have its OR time released is the one with the most unscheduled but allocated OR time [1,20].
Whenever possible, the OR manager should indeed put the case into the OR time of the service with the most allocated but unscheduled OR time. This is particularly important for long (> 3 hr) cases scheduled at medium (and small) surgical suites [20]. OR managers can reasonably compromise on releasing the OR time of the service with the largest difference between allocated and scheduled OR time when the new case is short (1 hr).
**c. Explanation at start of chapter 18: Day of Surgery Decisions (Table 2). The scenario in **Table 5 **is an example of application of the principle**.
On the day of surgery, OR efficiency is maximized by minimizing the hours of over-utilized OR time [1,11,19].

The reference numbers in brackets were changed to the reference numbers of this article from the reference numbers for the explanations. The references for the explanations are at the end of the document with hyperlinks to the abstract and full text. The underlining matches that seen in the explanations.

However, report generation is not possible for the increase in OR efficiency obtained by modifying case scheduling decisions before and on the day of surgery (Table 1). For example, a common cause of many prolonged turnovers in the middle of the workday is gaps between cases of different surgeons following each other in the same OR [2]. Statistical methods have been developed for quantification of the number of such prolonged turnovers by time of day and analyses of their impact on OR efficiency [1-3]. The automatic generation of these reports has been implemented for routine use [1,2]. In contrast, methods to identify the causes of the gaps have not been automated, because information systems generally do not record reasons that decisions were *not *made. For example, suppose a case was moved from OR 1 to OR 2 at 3 PM on a specific date. The room change would be documented in the audit trail. An assessment of the quality of that decision might be possible retrospectively, based on a reconstruction of the circumstances in every OR at the time [4]. However, this is often impractical, as illustrated by the following contrasting example in which a case was not moved. OR's 3-5 characteristically are used by the same specialty. A case started on time in OR 3 and finished during over-utilized time (Table 1) [1]. From the available stored data, it would appear that the case could have started earlier than scheduled in OR 4, thereby preventing at least some of the over-utilized OR time. However, the surgeon's assistant was unavailable, as she was occupied in OR 5, thus preventing the room change and the earlier start. Yet, relevant information about the availability of the assistant is not stored in the facility's information system. The fact that the decision not to move the case was an appropriate one cannot be construed from the available data, even in retrospect. The situation would appear as a gap in the schedule that could have been filled by another case to increase OR efficiency by reducing over-utilized OR time [5], but the case was not moved to fill the gap.

Assessment of these decisions is needed, because much of the work of the anesthesiologist or OR nurse manager running the control desk involves coordinating such restrictions on the availability of personnel and equipment [6]. Furthermore, decisions to move or not to move cases are often poor. The performance of anesthesiologists and nurses have been studied by using simulated scenarios that ask whether cases should be moved, as determined by the impact of the decisions on OR efficiency. Performance was worse than random guessing [7]. Both psychological biases [8,9] and lack of knowledge [10] contribute to these poor OR management decisions.

No systematic process has previously been described to assess decision-making policies and practices that influence OR efficiency at a health care facility [1] (Table 1). A practical process needs to be scalable so that it can be applied to multiple facilities within a health care system with no more human intervention than needed for a single facility. In this article, we describe the use of 1 hr structured meetings during which sufficient knowledge is elicited to assess the quality of OR management decisions influencing OR efficiency [1,7,11]. Approximately 45 scenarios (Table 2) from scientific articles are adapted (Table 3) automatically so that their cues are appropriate for each facility [2,12-14] (Table 4, 5). To clarify the organization of this article, conclusions from each section are listed in Table 6.

**Table 2:** Chapter Topics and Number of Scenarios in Each Chapter

Chapter	Scenarios	Title
1	3	Definitions - Service and Staffing^† ^
2	1	Definitions - Elective, Emergent, and Urgent
3	1	Definitions - Allocated OR Time
4	1	Definitions - Case Duration & Turnover Time
5	4	Definitions - OR Efficiency
6	1	Definition - Labor Productivity
7	2	Allocated OR Time versus Block Time
8	1	Definitions - Service and Allocated OR Time
9	4	Allocations to Maximize OR Efficiency
10	1	Allocating OR Time Based on Qualitative Data
11	2	Allocating OR Time to the OTHER Service
12	1	Estimating Total Under-utilized OR Time
13	1	Budgeting Based on Estimated Staffing^† ^Costs
14	5	Releasing OR Time Based on OR Efficiency
15	1	Scheduled Delays between Cases
16	6	Scheduling Cases to Maximize OR Efficiency
17	2	Moving Cases on the Day of Surgery
18	6	Day of Surgery Decisions
19	2	Sequencing Urgent Cases
		
	45	

^† ^Choosing staffing is synonymous with calculating optimal allocation of operating room (OR) time based on minimizing the expected inefficiency of use of OR time [1].

**Table 3:** Characteristics of Each of the Scenarios and Explanations

	**Scenarios**	**Explanations**
		
Number of scenarios	45	19
Number adapted	43	0
		
Adapted parameters		
Minimum	0	
25th percentile	3	
50th percentile	4	
75th percentile	6	
Maximum	9	
		
Pages in 16 point Arial		
Minimum	1	1
25th percentile	1	1
50th percentile	1	1
75th percentile	1	2
Maximum	1	2
		
Sentences		
Minimum	3	1
25th percentile	7	6
50th percentile	11	7
75th percentile	15	12
Maximum	22	24
		
Words		
Minimum	21	17
25^th ^percentile	69	86
50^th ^percentile	126	126
75^th ^percentile	158	238
Maximum	190	352

Percentiles are based on the characteristics of the 45 scenarios. The number of adapted parameters is independent of the number of times an adapted parameter is used in a particular scenario. For example, in Table 4, the parameter "[S2]" is used 5 times, but counts as only 1 adapted parameter. The numbers of sentences and words in each scenario are different ways of quantifying the lengths and heterogeneity of the scenarios. Each scenario fits on a single page.

**Table 4:** First of the 3 scenarios for which the described practice of the operating room manager of Hospital A did not match decision-making based on maximizing efficiency of use of operating room time

**4a. Adapted scenario printed on single page in landscape orientation using Arial 16 point font**		
	Among the 13 ORs typically started on Thursdays, there are 12 ORs allocated to specific services. The services "General Surgery," "Orthopedics," and "Pain Medicine" have each been allocated one OR. General Surgery has scheduled 9 hr of cases in its OR. Orthopedics has 6 hr of cases scheduled into its OR. Surgeons in Pain Medicine have scheduled a 3 hr case. The remaining ORs are fully booked. General Surgery wants to schedule another case.	
	OR management, staffing,^† ^and case scheduling decisions are made based on four ordered priorities: Safety, Access, OR efficiency, and Reducing patient waiting on the day of surgery.	
	General Surgery can book the case, because it has access to OR time on what ever work day the service chooses. If based on surgeon availability, staff knowledge, equipment availability, etc., the case can be performed safely in the OR time allocated for Pain Medicine, then Pain Medicine would have its OR time released for the General Surgery case. This is because Pain Medicine has the largest difference between the allocated and scheduled hours of cases (i.e., would be expected to have the most under-utilized OR time on the day of surgery).	
**4b. Scenario as stored in computerized library (line breaks added for clarity)**		
	Among the [S6] ORs typically started on [S5], there are [S1] ORs allocated to specific services. The services "[S2]," "[S3]," and "[S4]" have each been allocated one OR. [S2] has scheduled 9 hr of cases in its OR. [S3] has 6 hr of cases scheduled into its OR. Surgeons in [S4] have scheduled a 3 hr case. The remaining ORs are fully booked. [S2] wants to schedule another case.	
	OR management, staffing,^† ^and case scheduling decisions are made based on four ordered priorities: Safety, Access, OR efficiency, and Reducing patient waiting on the day of surgery.	
	[S2] can book the case, because it has access to OR time on what ever work day the service chooses. If based on surgeon availability, staff knowledge, equipment availability, etc., the case can be performed safely in the OR time allocated for [S4], then [S4] would have its OR time released for the [S2] case. This is because [S4] has the largest difference between the allocated and scheduled hours of cases (i.e., would be expected to have the most under-utilized OR time on the day of surgery).	
**4c. Unsolicited comments based on non-adapted scenario, shown with the corresponding stored parameters. None of the comments relate to the process of decision-making**.		
	S5, S6 We don't run 13 ORs on Thursdays	
	S1, S6 All of our ORs are planned for specific services every day	
	S2, S3, S4 We allocate "block" time by surgeon, not specialty	
	S2, S5 General Surgery doesn't do many cases on Thursdays	
	S6 We don't do pain medicine in our ORs, but at a clinic	
**4 d. Steps performed by software to adapt parameters S1 through S5 automatically**		
	1.	Using the most recent 9 four-week periods of data [1,12], calculated turnover times between each pair of sequential cases in the same OR on the same day. Turnover time was defined as time from when a patient exited the OR until the next patient entered the same OR on the same day [2]. Turnovers longer than 90 min were set equal to 90 min. The use of 9 periods is based on previous study using training-testing datasets, which showed that each increase in the number of four-week periods resulted in a statistically significant reduction in mean labor costs [12].
	2.	Calculated the OR workload for each service on each day [1]. The workload was defined as total hours of OR time and turnover times, excluding urgent cases [1]. Although the services were specialties for the specific hospital shown in the example, no such assumption was made [1].
	3.	Assigned each combination of service and day of the week with a mean OR workload less than 5.60 hr to the pseudo-specialty representing open, unblocked, first-scheduled, first-served "OTHER" time for low workload specialties (i.e., the service is not assigned its own OR) [1]. The value of 5.60 hr is the optimal break even point [13,14] based on over-utilized OR time costing 1.5 times as much as under-utilized OR time and OR workload by service and day of the week having a standard deviation of 0. Derivation can be shown by simple algebraic manipulation of equation (11) in Reference [21]. By Monte-Carlo simulation, results are insensitive to typical ranges of standard deviations [22].
	4.	For each combination of service and day of the week, the total inefficiency of use of OR time was calculated for choices of 0 ORs, 1 OR, 2 ORs, etc., and the choice with the smallest inefficiency was used as the allocation.
	5.	Tested the statistical assumption of randomness (e.g., no trend over time) as described in our review article [1], and if assumption violated, stopped and used non-adapted scenario for facility. Note that this situation has not occurred, as expected from the sample size chosen [1,12].
	6.	Set S1 to be the number of ORs allocated to individual specialties, excluding OTHER.
	7.	Determined which combinations of service and day of the week were allocated 1 OR.
	8.	Chose the earliest day of the week with at least 3 specialties allocated 1 OR. If situation did not exist, then scenario was not included in the collection of scenarios given to the facility. Otherwise, set S2, S3, and S4 to be the first 3 services allocated 1 OR for that day of the week, alphabetically. Set S5 to be the day of the week.
**4e. Automatic selection of S6**		
	9.	From among all cases performed during the most recent 9 four-week periods, selected those starting between 6:45 AM and 9:30 AM.
	10.	From among those cases, selected the first case of the day in each OR on each workday.
	11.	Calculated the median number of first case of the day starts separately for each day of the week.
	12.	Set S6 to be the number of ORs from step 11 on the S5 day of the week.

^† ^Choosing staffing is synonymous with calculating optimal allocation of operating room (OR) time based on minimizing the expected inefficiency of use of OR time.

**Table 5:** Second of the 3 scenarios for which the described practice of the operating room manager of Hospital A did not match decision-making based on maximizing efficiency of use of operating room time

**5a. Adapted scenario printed on single page in landscape orientation using Arial 16 point font**		
	At 12 noon, both OR 1 and OR 13 expect to be ready for their next patient in 45 minutes. Preparing each of the patients for surgery will take approximately the same amount of time.	
	Allocated OR time is from 7:30 AM to 5:00 PM. OR 1 is ahead of schedule by 30 minutes. OR 13 is behind schedule by 30 minutes. OR 1 is scheduled to end its cases at 7:00 PM. OR 13 is scheduled to end its cases at 5:00 PM.	
	Preparing which of the two patients should be a higher priority?	
	OR management, staffing,^† ^and case scheduling decisions are made based on four ordered priorities: Safety, Access, OR efficiency, and Reducing patient waiting on the day of surgery.	
	Maximizing OR efficiency (i.e., minimizing over-utilized OR time) is a higher-priority than reducing patient waiting from scheduled start times. Therefore, preparing the patient for OR 1 is a higher priority than for OR 13, even though OR 13 is behind schedule.	
**5b. Paragraphs 1, 2, and 5 as stored in computerized library (line breaks added for clarity)**		
	At 12 noon, both [S1] and [S2] expect to be ready for their next patient in 45 minutes. Preparing each of the patients for surgery will take approximately the same amount of time.	
	Allocated OR time is from [S3] to [S5]. [S1] is ahead of schedule by 30 minutes. [S2] is behind schedule by 30 minutes. [S1] is scheduled to end its cases at [S4]. [S2] is scheduled to end its cases at [S5].	
	Maximizing OR efficiency (i.e., minimizing over-utilized OR time) is a higher-priority than reducing patient waiting from scheduled start times. Therefore, preparing the patient for [S1] is a higher priority than for [S2], even though [S2] is behind schedule.	
**5c. Unsolicited comments based on non-adapted scenario, shown with the corresponding stored parameters. None of the comments relate to the process of decision-making**.		
	S1 OR 1 is being renovated	
	S2 We don't have an OR 13	
	S2 OR 13 is in a different building	
	S2 OR 13 patients are cardiac, they need more time	
	S3 We start the workday at 7:15 AM	
	S4, S5 Our workday is supposed to end at 3:30 PM	
**5d. Steps performed by software to adapt parameters S1 and S2 automatically**		
	1.	Using the most recent 9 four-week periods of data [1,12], calculated the ORs with the most cases and the second most number of cases. These are S1 and S2. Before using the names of the ORs, checked their names for inclusion of the terms "OR" in upper case or "rm" in either uppercase or lower case. If absent, added a preceding word "OR". The use of 9 periods is based on previous empirical study using training-testing datasets, which showed that each increase in the number of four-week periods resulted in a statistically significant reduction in mean labor costs [12].
**5e. Automatic selection of S4 and S5 (i.e., realistic times for ends of workdays in selected ORs)**		
	2.	Using the most recent 9 four-week periods of data [1,12], excluded cases that were urgent, performed Sunday through Wednesday, Friday, or Saturday. Thursdays are used for the automation because workweeks in different countries are Monday though Friday or Sunday through Thursday.
	3.	Identified the last case of the day in each OR on each Thursday, and for every Thursday counted the number of such last cases. Calculated 0.60 multiplied by that number of cases, where 0.60 is the optimal percentile based on over-utilized OR time costing 1.5 times as much as under-utilized OR time [21]. Derivation of optimality is shown on pages 313-316 of Reference [21].
	4.	Using the data from step 2, determined for each Thursday the earliest time at which a case exited from an OR while the number of still running ORs was less than the number of cases from step 3.
	5.	Took the median of the times from step 4. Set S5 to be the median rounded up to the next 15 minutes (e.g., 4:00 PM would be 4:00 PM whereas 4:01 PM would be 4:15 PM). However, before using it in the scenarios, the space between the numbers and the "PM" was changed to a non-breaking space.
	6.	Set S4 equal to S5 plus 2 hr, printed with a non-breaking space between the numbers and the "PM".
**5f. Automatic selection of S3 (i.e., realistic time for start of the workday)**		
	7.	Using the cases from step 2, excluded cases starting before 6:45 AM or after 10:00 AM.
	8.	From among those cases, selected the first case of the day in each OR on each Thursday.
	9.	Rounded each of the start times to the nearest 15 minutes. Created a histogram for the number of ORs among all Thursdays with the same rounded start times.
	10.	Set S3 equal to the most common time from step 9, printed with a non-breaking space between the numbers and the "AM".

^† ^Choosing staffing is synonymous with calculating optimal allocation of operating room (OR) time based on minimizing the expected inefficiency of use of OR time.

**Table 6:** Summary of Results of each section

Section 1: A process of event-based knowledge elicitation was developed by employing hypothetical scenarios to assess OR management decision-making at surgical facilities that may reduce the efficiency of use of OR time. The needs assessment is practical, occupying approximately 1 hour of local managers' time while they evaluate if their decisions are consistent with the described scenarios.
Section 2: Hypothetical scenarios addressing every OR management decision influencing OR efficiency were created from published examples.
Section 3: Scenarios are adapted, so that cues about conditions are accurate and appropriate for each facility (e.g., if OR 1 is used in a scenario, the listed procedure is a type of procedures performed at the facility in OR 1).
Section 4: For 43 of 45 scenarios, adaptation is performed automatically using Visual Basic for Applications code and the facility's OR information system or AIMS data.
Section 5: Facilities consistently needed to make few changes in decisions to increase the efficiency of use of OR time. However, based on 22 applications of the process, there are differences among facilities. Thus, the needs assessment should be performed individually for each hospital.
Section 6: A table of contents of the indexed scenarios is created automatically, providing a simple version of problem solving using case-based reasoning. For example, a new OR manager needing to know how best to decide whether to move a case can look in the chapter on "Moving Cases on the Day of Surgery" (Section #4) to find a scenario that describes the situation being encountered.

The underlined verb in each Section shows the specific Result, emphasizing that each of the listed conclusions is limited in scope to that shown from the limited data presented in the paper. These listed conclusions match those in the paper's Abstract. "OR" represents operating room. "AIMS" represents anesthesia information management system.

## Methods and Results

### 1. Review of event-based knowledge elicitation

Knowledge elicitation is the process of collecting information from a human source [15]. The purpose of using scenarios dealing with OR management is to elicit knowledge from managers about how they would respond under given circumstances. For example, by using the adapted scenario of Table 4a, knowledge was elicited from members of the OR management team at a hospital referred to as Hospital A about how they handle the release of allocated OR time. The scenario is 1 of 5 scenarios about the release of allocated OR time, each adapted to elicit different information (Table 2 row 14) [1,16].

This process is an example of "event-based knowledge elicitation," wherein human sources of knowledge (i.e., local OR managers) are provided with known and controlled scenarios. They then report what their decisions would be under the circumstances provided [17]. The scenarios all describe desirable decisions that increase OR efficiency and/or reduce patient waiting, while not compromising safety. For each scenario, the managers report concordance or discordance of their decision-making with the decision described in the scenario (Table 2). A phone or web conference facilitates review of the scenarios as the anesthesiologist and OR nurse manager page through them. A consultant moderates the conference that typically lasts 1 hour.

Generally, event-based knowledge elicitation is appropriate when the objective is to obtain information from many individuals in different organizations to compare decisions to a standard [17]. Event-based knowledge elicitation is a test-like process. It is appropriate for evaluating OR management decisions, likely more so than for operational decisions in other healthcare areas such as clinics and emergency departments. Binary decisions (e.g., move or do not move the case) are compared to the standard of whether the decision is likely to increase the expected efficiency of use of OR time (Table 1) [18-20]. This standard is appropriate regardless of the health care system or model of professional compensation, subject to the reasonable assumptions that only one patient is in each OR at a time and that surgery is non-preemptive [1,11,21-23]. Non-preemptive means that surgery cannot be stopped in the middle and completed the next day, like an airplane flight cannot be interrupted in the middle. Event-based knowledge elicitation results in a needs assessment that identifies the changes in decision-making necessary to achieve the maximum possible increase in OR efficiency.

Event-based knowledge elicitation has two main drawbacks. First, some scenarios may not be adaptable using the archived data obtained from facilities [24]. This limitation is addressed in Section 4 below. Second, substantial time is required initially to generate scenarios that elicit the appropriate information and can be adapted automatically [17]. The set of scenarios must assess all relevant aspects of important decisions associated with management practices that influence OR efficiency (i.e., have content validity, as considered in the next section).

### 2. Examples of scenario creation and adaptation

Table 2 lists the topics of the 45 hypothetical scenarios used for knowledge elicitation. The scenarios were created from published examples of decisions that increase OR efficiency [1,7,11,14,16,19,20]. References [1] and [11] are peer-reviewed comprehensive reviews of decision-making before and on the day of surgery. Table 2 lists the 19 OR management topics covered. Content validity was established by their correspondence to a complete set of decisions (i.e., those of References [1,11]) that influence the efficiency of use of OR time (e.g., allocation of OR time, scheduling of cases, scheduling of add-on cases, and moving of cases).

Table 3 gives the characteristics of the scenarios (e.g., to illustrate their complexity). Among the 45 scenarios, there are 0 to 9 adapted parameters (25^th ^percentile = 3, 50^th ^percentile = 4, and 75^th ^percentile = 6 adapted parameters).

Table 4a is an adapted scenario describing a decision about releasing allocated OR time. The scenario is similar to that published in Reference [1] (page 1510 column 1). However, its parameters have been adapted to match those of Hospital A. The scenario considers releasing allocated OR time based on increasing the efficiency of use of OR time [1,20]. The scenario deliberately contains three services rather than two services to emphasize prior findings that the expected inefficiency of use of OR time is increased substantially by selecting the wrong service to have its OR time released [20]. Service refers to the unit used for case scheduling, typically specialty. The scenario purposely does not specify the current weekday or the number of days before the date of surgery. The reason is that OR efficiency is increased by releasing OR time based on a threshold rule that decides if the case would otherwise be scheduled into over-utilized OR time, not the common practice of releasing time a prespecified number of days before surgery [16]. Frequent problems with releasing time arise from poor statistical forecasting of workload by service based on day of the week, not from releasing allocated OR time too many or too few days in advance [1,20].

Table 4b is the template for the scenario that shows how the scenario was adapted systematically. The non-adapted scenario is stored in an Office Excel file (Microsoft, Redmond, WA). Line breaks were inserted into the template in 3b to highlight parallelisms between the adapted scenario in 3a and the computerized version in 3b. The values in square brackets "[S1]", "[S2]", ... "[S6]" are parameters that are replaced automatically with appropriate values adapted for the individual facility using Visual Basic for Applications (VBA) computer code and OR information systems or AIMS data.

Table 4c shows examples of non-solicited comments on allocation and release of OR time received from facilities prior to use of adapted scenarios. Teaching and lectures also evoked such comments. For each, the relevant parameter is shown (e.g., S1), since the comments were used to adapt the scenarios so that each local OR manager would see values appropriate for his or her facility (e.g., as in Table 4a). The "surface properties" of the adapted scenario were matched.

Table 4 sections d and e show how data from a facility are analyzed automatically to determine values of the parameters in the template that customize the scenario. The parameters are calculated from OR information systems or AIMS data (i) to save time for consultants and (ii) to reflect actual practice, not the facility's rules for what is supposed to happen. For example, the number of first case starts on the day of the week chosen in Table 4d is obtained from the steps in Table 4e. Similarly, the actual time of the start and end of the regularly scheduled workday for the scenario of Table 5a are obtained from the steps in Table 5e and 5f. As another example, some surgeons usually work in only a few specific ORs due to the presence of specialized equipment in those rooms. That information is fully captured by the adaptation when it chooses a surgeon and then selects an OR based on those ORs in which the surgeon frequently works. None of the data used are protected health information.

### 3. Rationale for adapting scenarios ("cues")

When an OR manager makes decisions for a facility, the particular OR and surgeon involved often serve as sources of information ("cues") about conditions [25] that will limit the decision options.

For example, consider a hospital with a retina surgery program in which OR 1 has the only ophthalmological microscope, and it is ceiling mounted. At 1:00 PM, the OR charge nurse receives a phone call, hangs up, and says to the anesthesiologist "add-on OR 1." That piece of information (i.e., "cue") alone is sufficient to let the anesthesiologist know that the nurse is referring to an add-on retina case. "OR 1" is said to be a "surface condition" of the scenario.

Many facilities use the same names to label ORs (e.g., OR 1 and OR 2), and these names are not interchangeable among facilities. Whereas a non-adapted prototypical scenario may refer to a retina case in OR 1, OR 1 may be a trauma room at the hospital where the scenario is being considered. The name of the OR thus provides a cue to the type of surgery being performed there, the surgeons, the equipment, etc. This can create confusion if scenarios are not adapted to the cues at each facility. Local OR managers may otherwise interpret cues incorrectly and assign them an unintended meaning. The managers make seemingly illogical decisions, not even realizing the misunderstanding (see Table 4c and 5c). Cues must be included in scenarios, because individuals *appropriately *base their decisions on cues. Unless the cues are included, elicited decisions are unrealistic or require an inordinate amount of thought.

Scenarios are adapted for each facility (Table 4 and 5) because individuals with substantial experience about their own facility rely on cues for decision-making when they lack broad conceptual [26] knowledge. Our impression is that experts (researchers) in the science of OR management evaluate decisions differently when unaware of local conditions. After hearing the first 1-2 sentences of the scenario, experts classify the scenario based on the type of decision (e.g., "case scheduling decision before the day of surgery" [1,19] versus "moving cases on the day of surgery" [11,27-30]). Then, knowing what data will be needed, they listen for conditions that will limit the ORs in which a case can be done, when the surgeon will be available, etc. Experts do this using their stronger conceptual knowledge of the rationale for good decision-making and a hierarchical set of goals and conditions under which decisions should be made [11,25].

Our perceptions of the importance of cues and how local managers assign meanings to them could be biased because our impressions are based on the non-random sample of OR managers with whom we have worked. Thus, experimental studies were examined that compare how novices (e.g., undergraduate students) and experts solve problems that have unique correct answers to help us understand the foundation for differences between managers and experts in their approaches to decision-making [31,32].

*Experimental Study 4A*) Advanced graduate students and novice undergraduates were given cards with scenarios describing basic physics problems [31]. Each participant sorted the cards based on the similarity of the problems. Card sorting is a common method of knowledge elicitation [24]. The graduate students clustered the scenarios based on conceptual knowledge (i.e., physical principles such as Law of Conservation of Energy) [31]. Novices clustered the scenarios based on surface content (e.g., two scenarios with springs were perceived as similar) and based on keywords (e.g., two scenarios with the word "friction" were perceived as similar) [31].

*Experimental Study 4B*) Mathematicians, undergraduates taking an introductory college mathematics course, and undergraduates taking an introductory computer programming course were given cards with mathematical scenarios [32]. The mathematicians sorted the cards based on conceptual principles [32]. Undergraduates taking the programming course sorted based on surface conditions [32]. The performance of mathematics course participants was significantly closer to that of the mathematicians [32].

Our impressions of OR managers and the importance they place on cues matches the experimental results showing that cues are much more important to novices.

Another experiment demonstrated the importance of adapting the scenarios for knowledge elicitation [33,34].

*Experimental Study 4C*) Undergraduates were given worked sample problems in statistics (probability), and then test problems. For some participants, the test problems had the same parameters as the sample problems. Other participants were given test problems with different parameters. The latter participants scored significantly worse [33,34]. Confidence in the answer was also lower when different parameters were substituted [33]. Even when the story was also changed, switching of the parameters degraded learning [34]. When the parameters were similar between the sample problems and the test problems, the parameters acted as a cue to help the students recall relevant principles.

Given that each manager spends 1-2 minutes per scenario, the scenarios must be carefully worded to elicit the correct answer (i.e., the decision they would make in practice). Experiment C shows that the surface properties of the scenarios (i.e., the adapted parameters) are important for keying the managers into the actual situations they encounter.

The experimental studies also highlight an analogy between our use of adapted scenarios and "case-based reasoning," in which knowledge is stored in the form of cases of lengths typically less than 200 words (Table 3). Case-based reasoning is not the same as "case-based learning" (e.g., as used in business or medical school cases). Materials for case-based learning are long descriptions from multiple perspectives and are addressed by students over several hours [35]. Correct answers cannot be discerned simply by finding an analogy to a case that has been solved.

### 4. Process of adapting scenarios to have cues match local values

The application of case-based reasoning to eliciting OR management decisions revolves around the process of selecting scenarios for a facility and then adapting them. The OR information systems or AIMS data obtained for adaptation of the OR management scenarios include the names of the services, and then for each case: OR in which case was performed, date/time into OR, date/time out of OR, urgent or not, and surgeon. Holidays and equivalent "slow-down" days are inferred automatically from days with very low workloads [1]. For Table 4 and 5, specific ORs and service names were altered for publication to maintain confidentiality.

Generally, developing a "case-based reasoning" system is time consuming because detailed domain (application) knowledge (e.g., ability to distinguish between important cues and irrelevant surface conditions) is needed. Such knowledge must be developed, then translated into computer code that will adapt the scenarios automatically to include cues matching local values [36]. The complexity of the task for generating OR management scenarios is evidenced by the steps in Table 4d, 4e, 5d, 5e, and 5f.

Another characteristic of case-based reasoning systems is progressive improvement in the library of scenarios [36]. After each of the 22 times that a new set of scenarios was created for facilities, small iterative improvements were made in the computer code.

Two of the 45 scenarios are not currently adapted for any facility because of limitations in the data obtained (Table 3) (see above Section 1). One non-adapted scenario involves a definition of the difference between assignment, staff scheduling, and allocated OR time. The scenario refers to anesthesiologists and medical direction of anesthesia residents at an academic medical center. It is not currently adapted because information on the type of anesthesia provider is not available for each case. The scenario was therefore written without specific names of ORs to avoid providing unintentional misleading cues. The other scenario not adapted involves an example of the difference between urgent and elective cases. Surgical procedures are not obtained for each case and thus representative urgent and elective procedures cannot be chosen. Even if procedures were available, most facilities having us perform knowledge elicitation do not schedule cases using a systematic vocabulary (e.g., Current Procedural Terminology codes) [36]. The data currently obtained are limited to those listed in Table 4d-e and 5d-f.

### 5. Examples of use of the adapted scenarios by surgical facilities

Adapted scenarios have been created for 22 facilities to identify deficiencies in OR management that compromise the efficiency of use of OR time. A separate needs assessment was necessary for each facility because results were not consistent among facilities. Data from the most recent three analyses performed in 2009 are presented below. The three private non-academic hospitals are from separate health systems in different US states, and are designated "Hospital A," "Hospital B," and "Hospital C.". The number of ORs at the hospitals are the 50^th^, 90^th^, and > 95^th ^percentiles among US surgical facilities [38].

The adapted scenarios and explanations were presented as an Adobe Acrobat PDF document for which page deletion was permitted. The document was titled "Needs Assessment." During a 1-hour web conference, several managers from each facility reviewed each scenario and deleted each page (and corresponding explanations) for which the scenario matched their routine practice (see section 1). Deleted scenarios were not reviewed further because those processes were not contributing to the inefficiency of use of OR time.

Each PDF report included a table of contents, 41 or 43 scenarios, 19 explanations, and a bibliography with hyperlinks to articles. Hospitals A and B both had 43 scenarios out of the available 45 scenarios (Table 2 and 3), but 2 scenarios did not apply. For example, Hospital A had one scenario that did not apply to Hospital B, because at B the "OTHER" service was not allocated precisely one OR on any weekday. By OTHER, we mean the first-come first-scheduled unblocked open overflow virtual service. Hospital B had one scenario that did not apply to Hospital A, because at A there was no service allocated OR time on some but not all days of the workweek. The allocations were calculated based on maximizing the efficiency of use of OR time (see Table 4) [1,7,11-14,16,19-22,30,39]. Hospital C received both of the scenarios excluded from Hospitals A and B. Three of four scenarios were excluded for Hospital C because no service except for OTHER was allocated more than one OR on any weekday. Knowing which scenarios were excluded for each facility provides insight useful for the creation of a policy manual because the corresponding decisions are irrelevant to the facility and thus do not need to be part of their manual. The fact that hospitals differ with respect to the specific scenarios that are included means that the table of contents must be (and is) generated automatically. The table of contents facilitates use of the collection of scenarios as a policy manual (see below Section 6).

Of the 41-43 scenarios, only 3-5 did not match decision-making at each hospital. Table 4 and 5 show 2 of 3 such scenarios for Hospital A. The other adapted scenario involved moving cases based on reducing expected over-utilized OR time [1,11,19,28]. Thus, the deficiencies in daily decision-making by the local managers that resulted in unnecessary over-utilized OR time involved three types of situations: choosing services to have OR time released, moving cases, and calling for next cases in ORs. For Hospital B, the scenario in Table 5 was considered different from current practice, as well as two scenarios on allocation of OR time based on maximizing OR efficiency. Hospital B had been focusing its educational and management efforts almost entirely on physician decision-making on the day of surgery and on choosing which surgeons should be assigned first case starts (i.e., surgeon-specific block time). The facility learned instead that OR allocations and calling for patients needed modification. For Hospital C, one of the five retained scenarios was that of Table 5. Two scenarios related to "Day of Surgery Decisions" based on medical direction of nurse anesthetists by anesthesiologists to reduce over-utilized OR time or, when over-utilized OR time did not occur, to reduce expected tardiness from scheduled start times [11]. Two other scenarios dealt with "Sequencing Urgent Cases" [11]. Strikingly, the excess inefficiency of use of OR time at Hospital C was attributable almost solely to decision-making on the day of surgery, unlike for Hospitals A and B. Excess anesthesia labor costs [1] from poor OR allocation and case scheduling ranged from 20% to 30% among the three facilities. The scenarios were thus successful in identifying deficiencies that compromised the efficiency of use of OR time at these facilities. Results were all consistent with separate quantitative analyses [1] of the hospitals' OR allocations, turnover times, etc., performed using the methods described in McIntosh et al. [1], indicating concurrent validity.

### 6. Formatting and indexing the scenarios for use as the facility's policy manual

Because most of the decisions (e.g., 95%) elicited from the scenarios coincided with evidence-based decisions, recognition of the few deficiencies was particularly useful. When scenarios were identified for which the hospital's decision-making did not match that which maximized OR efficiency, the adapted scenarios served as training materials (e.g., part of the facility's revised policy manual).

The scenarios that comprise the policy manual form a computerized library that is indexed at two levels. The upper level is represented by the table of contents ("chapters") of the policy manual created for the facility. For example, one chapter is "Definitions - Case Duration & Turnover Time" and another is "Moving Cases on the Day of Surgery". The lower level reveals the individual scenarios within each chapter. There are 1 to 6 scenarios per chapter (Table 2).

A chapter title from Table 2 is printed as a header at the top of each page from that chapter, with the numbering of the retained scenarios for the chapter in parentheses. Each scenario is one page long (Table 3). For example, one chapter has four scenarios, with titles "Definitions - OR Efficiency - Scenario (1)" through "Definitions - OR Efficiency - Scenario (4)." Although the purpose of the chapter titles is to facilitate use of the scenarios for case-based reasoning (see Section 4 above), the value of this feature cannot practically be evaluated with local OR managers, because each facility characteristically has only 2 or 3 such managers, with backgrounds that are not homogeneous [38]. The fourth experimental psychology study reviewed shows that pooling of results would likely be invalid [9,39,40]:

*Experimental Study 7D*) Undergraduate psychology students were given three scenarios with solutions ("worked problems") about basic probability theory, then answered test questions [40]. Titles in the experiment served as cues to indicate that new problems were addressing similar goals as the worked problems [40]. Even when the titles were not meaningful, students used the labels to help identify relevant problems [40]. Therefore, we conclude that the titles above the OR management scenarios facilitate the grouping of scenarios into cohesive topics relevant to OR management, even though local managers generally lack the corresponding conceptual knowledge [10] to understand the groupings.

Titles help readers identify related topics [40,41].

Each chapter includes an explanation to help managers with limited conceptual knowledge of OR management science to understand the rationale that underlies good decision-making. Table 1 gives several examples of explanations. For example, the briefest explanation in Table 1c is for "Day of Surgery Decisions" (Table 2) and applies to the adapted scenario of Table 5. Again, because each facility has few managers and facility conditions differ, we cannot practically test the usefulness of explanations with OR managers. However, an experimental study suggests [42] that those OR managers who have been unknowingly making suboptimal decisions for years are more likely to apply knowledge when there are explanations justifying [43] decision-making in relevant scenarios:

*Experimental Study 7E*) To study the value of explanations, high-school science students were given examples of the correct application of physics principles for which they had misconceptions [42]. The students continued to answer test questions incorrectly [42]. The students understood how the examples applied to the test questions, but they essentially refused to accept the implications [42]. Explanations providing a strong conceptual knowledge of the principles were necessary for correct problem solving.

Education in the science of OR management, including detailed explanations and examples, increased student (including OR managers) "trust in applying evidence-based statistical methods and analytic reports in healthcare management decisions" [44].

Explanations are generally not as useful as examples [45,16,47,48]:

*Experimental Study 7F*) Undergraduate psychology students were challenged with problems, and provided with an example solution and either a correct or an incorrect explanation of the solution [45]. The problems were ones that the students had not seen previously. The explanations had no influence on test responses, showing that the students ignored the explanations in lieu of the examples [45]. Students preferred examples to explanations even when the examples were brief and the explanations were detailed [45]. In another experiment, undergraduates given algebra word problems had higher post-test scores when provided with a simple example and a complex example versus either a simple example alone or a simple example and an explanation of the solution [46]. The algebra questions [46] were nicely relevant to add-on case scheduling decisions [11,47,48]: "Ann can type a manuscript in 10 hour and Florence can type it in 5 hour. How long will it take them when they can both work together?"

Thus, at least one scenario is available to serve as an example for each operational decision influencing OR efficiency, and the median is 2 scenarios (Table 2). Since each scenario is indexed, it can be retrieved for use in any situation using the cues that the situation provides. The process is as simple as inquiring about moving cases, and looking in the chapter on "Moving Cases on the Day of Surgery" (Table 2).

## Discussion and Conclusions

Our conclusions and their implications are listed in Table 6.

One limitation is that the process of knowledge elicitation identifies problems, but does not solve them. Other resources generally are necessary to implement the necessary changes. For example, from Section 5, the three most recent needs assessments for 2009 all identified that the facilities were not calling for the appropriate next patient preferentially from ORs with over-utilized OR time. They were not doing so even though one of the objectives of each facility was to reduce costs by reducing over-utilized time (Table 1). Knowing when to call for the next patient relies on knowing the expected over-utilized OR time in each OR, which in practice requires predicting the time remaining in on-going cases [48]. Recent work describes how to automate this process [48,49].

Another limitation is that the experimental studies reviewed in this article include systematic reviews of relevant experimental articles from two disparate fields, knowledge engineering (i.e., "event-based knowledge elicitation" and "case-based reasoning") and science education. These reviews were included to provide a foundation for our use of scenarios and our organization of the policy manual. However, relevant scientific information from experimental studies is limited. The knowledge engineering field generally addresses areas in which there are few experts worldwide. An expert is able to develop methods to elicit knowledge and transfer it to many users of the science. A consequence of having few experts in any field is that there are few experimental or observational studies quantitatively evaluating the performance of different knowledge elicitation methods. Most articles are principally qualitative reports describing development of a novel system. Another limitation of the experimental results is that their applicability to OR managers is unknown. Undergraduates are usually used as subjects. They are not practicing managers, but have the advantage that many subjects with similar backgrounds can be given the same problems. The types of problems given to the undergraduates apply well to our study because both the problems and the OR management topics in our scenarios have optimal mathematical solutions. Results seem applicable to OR managers because our qualitative observations of OR managers are consistent with experimental results.

## Competing interests

The University of Iowa performs statistical analyses for hospitals and anesthesia groups, including the reports described in the current manuscript. FD and REW receive no funds personally other than their salaries from the University of Iowa, including no travel expenses or honoraria, and have tenure with no incentive program.

RHE is President of Medical Data Applications, Ltd., whose CalculatOR™ software includes the analyses considered in this article.

## Authors' contributions

FD created the scenarios, wrote some of the computer code for adaptation, performed all of the consultations, designed the study, conducted the qualitative literature review of experimental studies, and wrote the initial manuscript. REW designed the study, expanded upon the literature review, and substantially modified the manuscript. RHE modified the scenarios and wrote most of the computer code for adaptation. All authors read and approved the final manuscript.

## Pre-publication history

The pre-publication history for this paper can be accessed here:

http://www.biomedcentral.com/1472-6947/11/2/prepub
